# Photochromic Donor–Acceptor Naphthalenediimides Enabling Near‐Infrared Photothermal Conversion for Seawater Desalination

**DOI:** 10.1002/chem.71482

**Published:** 2026-07-31

**Authors:** Sotaro Kusumoto, Soushi Kurihara, Masaya Shimabukuro, Naoki Ogiwara, Nobutsugu Hamamoto, Jack Harrowfield, Yang Kim, Yoshihiro Koide

**Affiliations:** ^1^ Department of Applied Chemistry Faculty of Chemistry and Biochemistry Kanagawa University Yokohama Japan; ^2^ Laboratory for Biomaterials and Bioengineering, Institute of Integrated Research Institute of Science Tokyo Tokyo Japan; ^3^ Center of Advanced Instrumental Analysis Kyushu University Kasuga Fukuoka Japan; ^4^ Graduate School of Science and Technology for Innovation Yamaguchi University Yamaguchi Japan; ^5^ ISIS Université De Strasbourg Strasbourg France; ^6^ Department of Chemistry Graduate School of Science and Technology Kumamoto University Kumamoto Japan

**Keywords:** naphthalenediimide, photochromism, photothermal conversion, water desalination

## Abstract

Photochromic donor–acceptor molecules that produce near‐infrared (NIR) absorption upon light activation offer promising platforms for organic photothermal materials. Here, neutral naphthalene diimide (NDI) derivatives with tertiary amine substituents demonstrate efficient photothermal conversion through photochromism‐induced radical formation. UV irradiation initiates intermolecular electron transfer from the electron‐donating amine groups to the electron‐deficient NDI core, generating stable radical species with strong NIR absorption. These photoactivated states enable rapid photothermal heating under 850 nm light with efficiencies reaching up to 24.5%. When integrated into a floating evaporation platform, the optimized compound achieves a water evaporation rate of 0.98 kg m^−^^2^ h^−^^1^ and an energy conversion efficiency of 65.5% under 1 sun illumination. These findings highlight the potential of photochromic NDI donor–acceptor systems as adjustable organic photothermal materials for solar‐driven water purification.

## Graphical abstract

Photoinduced radical formation in donor–acceptor NDI molecules generates strong near‐infrared absorption, enabling efficient photothermal conversion and solar‐driven seawater desalination.

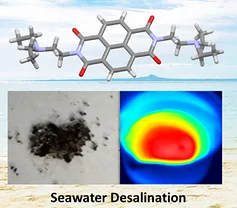

## 1 Introduction

Solar radiation is regarded as a clean and renewable energy source, and has been extensively exploited in photochemical reactions [1, 2], photovoltaic equipment [3, 4], photothermal conversion [5, 6, 7, 8], and photocatalysis [9, 10, 11, 12] as an environmentally friendly means to address global energy shortages. Among these applications, thermal conversion of solar radiation, as a heat source for water evaporation [13, 14, 15, 16, 17, 18, 19, 20], has recently attracted increasing attention as a sustainable approach to purifying water from limited or low‐quality sources. For the development of efficient materials, it is crucial to explore systems that exhibit broad solar radiation absorption, chemical and thermal stability, photostability, water insolubility, and high photothermal efficiency [21].

Materials developed to convert solar energy into heat include inorganic materials [22, 23, 24], carbon‐based materials [25, 26], organic small molecules [27, 28], and conjugated polymers [29, 30]. Organic small‐molecule systems, in particular, offer advantages such as structural tunability and high efficiency through molecular modification. However, their synthesis is often complex, leading to high costs and difficulties in large‐scale production. Consequently, their use has been mainly explored in biomedical photothermal therapy [31, 32, 33], while applications in solar‐driven water evaporation, such as seawater desalination, remain underexplored.

Photochromic molecules are promising candidates for photothermal conversion due to their simple synthesis and broad light absorption in their photoinduced state [34, 35]. Viologen derivatives, known for their strong electron‐accepting properties, have been widely studied for their photochromic behavior [36, 37, 38]. Although many molecular viologen salts are ionic and often water‐soluble, their solubility can vary substantially depending on their molecular structures, polymerization states, and counteranions. Therefore, photochromic materials with low water solubility and strong absorption extending into the near‐infrared (NIR) region are more desirable for solar evaporation applications.

Naphthalene diimide (NDI) derivatives with alkylamino groups, which contain both donor and acceptor sites within a single molecule, have been reported to show photochromism and, in some cases, NIR absorption, depending on the donor groups [39]. In addition to such molecular systems, NDI‐based coordination polymers [40] and NDI‐containing organic–inorganic hybrid materials [8] have been reported to exhibit radical‐anion‐derived NIR absorption and photothermal conversion. However, their application to solar‐driven seawater evaporation has rarely been investigated.

In this study, we present the use of a readily synthesized, water‐insoluble, photochromic small NDI‐derived molecule as a photothermal conversion material with near‐infrared absorption and evaluate its potential for solar‐driven seawater evaporation.

## 2 Results and Discussion

### 2.1 Structural Characterization and Photochromism

Compounds **1**–**3** were synthesized from naphthalene‐1,4,5,8‐tetracarboxylic dianhydride and the diamines: *N,N‐*dimethyl‐1,3‐propanediamine (for **1**), *N*,*N*‐diethylethylenediamine (for **2**), and 1‐(3‐aminopropyl)pyrrolidine (for **3**) [14]. The presence of one tertiary center on each diamine assured the formation of simple di‐imides only. Their identities were confirmed by elemental analyses and comparison of their powder x‐ray diffraction (PXRD) patterns (Figure S1) with those simulated from single‐crystal x‐ray diffraction (SCXRD) data [39].

The crystal structures of **1**, **2**, and **3** have been reported by Matsunaga et al. in the context of solid‐state photochromism [39]. In the present study, we briefly revisit these structures to highlight the packing environment relevant to the photoinduced optical and photothermal properties. Crystals **1**–**3** consist of one‐dimensional slipped stacks formed by π–π interactions between the planar NDI cores along the *a*‐axis (Figures 1a–f and S2). The adjacent NDI cores form π–π stacked arrangements in **1**–**3** (Figure 1d–f). The centroid‐to‐centroid distances between adjacent NDI cores were determined to be 3.604 for **1**, 3.671 Å for **2**, and 3.652 Å for **3**, respectively (Figure S3). In all cases, the interplanar angle between neighboring NDI cores is approximately 0°, indicating essentially parallel stacking of the NDI planes. In addition, short intermolecular C⋯O contacts involving the imide carbonyl groups were observed in the range of 3.188–3.276 Å (Figure 1d–f), and the tertiary amine nitrogen atoms are positioned near the electron‐deficient NDI cores of adjacent molecules, with N⋯C distances of 3.857–4.786 Å (Figure S4). Electrostatic potential (ESP) surface maps also show relatively electron‐rich tertiary amine moieties and electron‐deficient NDI cores, supporting the donor–acceptor assignment of these molecular components (Figure S5). Although these N⋯C separations alone should not be regarded as direct evidence for efficient through‐space electron transfer, they indicate the proximity of the amine donor units to the NDI acceptor framework. Together with the closely stacked NDI arrays and short carbonyl C⋯O contacts, these packing features are consistent with those previously discussed in relation to solid‐state photoinduced NDI radical‐anion formation and radical‐anion/mixed‐valence stack formation in these compounds [39]

**FIGURE 1:**
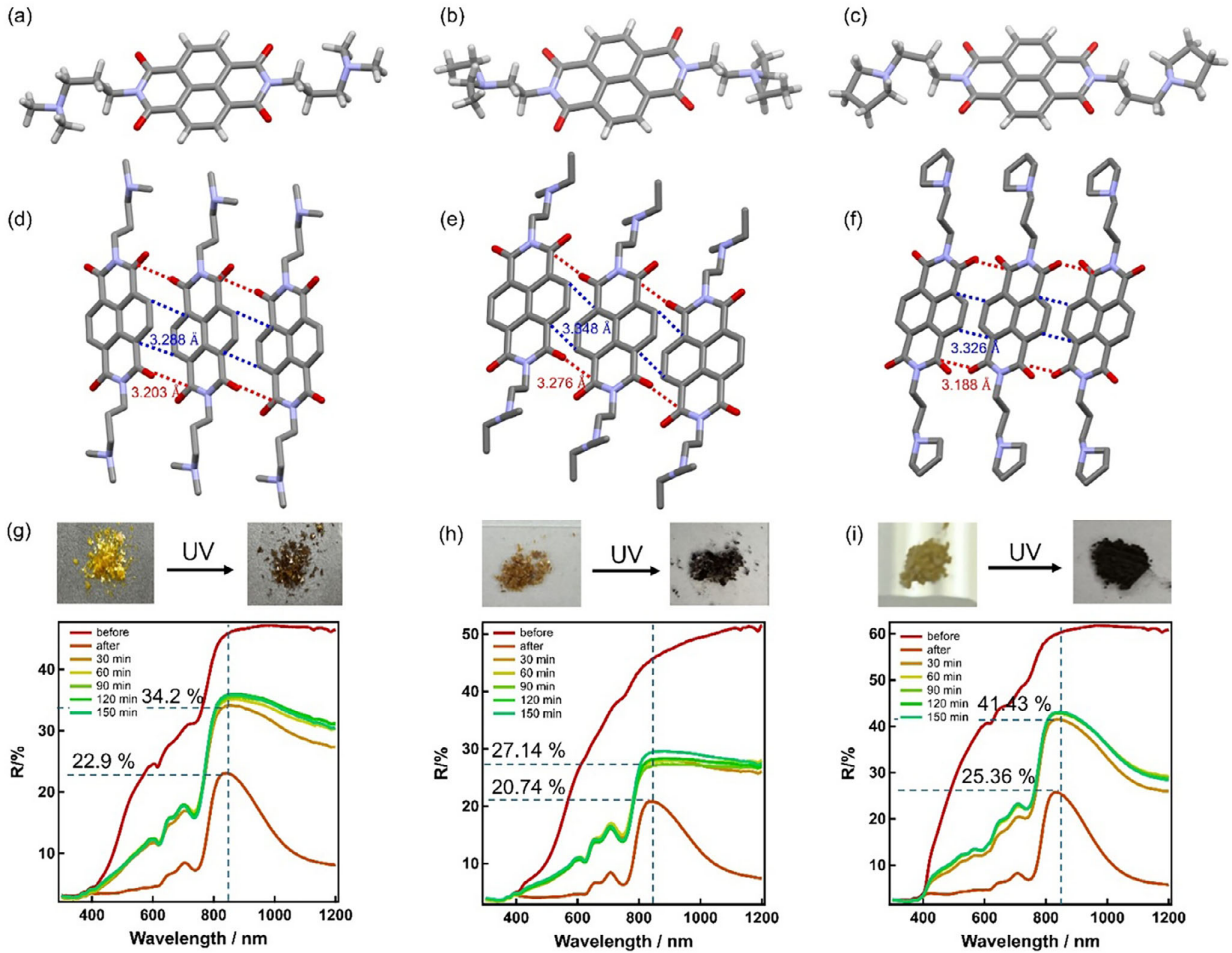
Crystal structures of **1** (a), **2** (b), and **3** (c). Intermolecular interactions between adjacent planar cores (red dashed line: C⋯C distance; blue dashed line: C⋯O distance) in **1** (d), **2** (e), and **3** (f). Photochromic behavior and time‐dependent diffuse reflectance of **1** (d), **2** (e), and **3** (f).

The yellow powders of **1**–**3** turned dark brown/black (Figure 1g–i) upon UV irradiation (*λ* = 365 nm, 0.03 W cm^−^^2^) for 5 min. Consistent with the color change, diffuse reflectance UV–vis–NIR spectra showed the emergence of broad absorption bands extending from the visible to the near‐infrared region after UV irradiation (Figure S6). To experimentally verify the formation of radical species, ESR measurements were performed (Figure S7). A weak ESR signal was observed even before UV irradiation, likely due to a small number of radicals formed during sample handling under ambient light. Upon UV irradiation, the ESR signal intensity increased significantly, confirming the photoinduced generation of radical species. These results indicate that the UV‐induced color change is associated with radical formation. To rationalize the photoinduced electron‐transfer process, DFT calculations were then performed. The calculated VBM is mainly distributed over the electron‐rich tertiary amine groups, whereas the CBM is localized predominantly on the electron‐deficient NDI core (Figure S8), supporting photoinduced electron transfer from the tertiary amine groups to the NDI core. To assess the durability of the radical, diffuse reflection spectra were recorded every 30 min over 2.5 h after UV irradiation (Figure 1g,h). The overall reflectance decreased during the first 30 min, with measurements at 850 nm showing 22.9% for **1**, 20.74% for **2**, and 25.36% for **3**. When the samples were then exposed to ambient light for another 30 min, the reflectance increased to 34.2% for **1**, 27.14% for **2**, and 41.43% for **3**. These changes indicate that UV irradiation induces broad absorption extending into the NIR region, while subsequent exposure to ambient light partially bleaches the photoinduced absorption. Upon UV irradiation, photoinduced electron transfer from the amine moieties to the NDI units generates NDI radical anions and amine radical cations. Although isolated NDI radical anions generally show limited absorption in the NIR region above 800 nm [41], the NDI units in compounds **1–3** are closely packed in the crystalline state. Therefore, the photogenerated NDI radical anions can electronically interact with adjacent NDI units or radical anions to form π‐radical aggregate states and/or intermolecular charge‐transfer‐like states [42]. These solid‐state electronic interactions give rise to lower‐energy electronic transitions, resulting in the strong NIR absorption observed for compounds **1–3**. Further measurements showed only minor changes in reflectance, and the dark brown/black color did not fully revert to the original yellow under ambient conditions. In addition, reflectance did not fully recover even after 1 month (Figure S9). This behavior should not be interpreted as indefinite stability of the initially generated radical species. Rather, given that these compounds were previously reported to recover their original color when stored in the dark [39], the incomplete recovery observed under ambient conditions is likely attributable to photoactivation by ambient/natural light. Thus, the radical‐related absorption is considered to be dynamically maintained through a balance between photoinduced radical generation and radical decay or transformation.

### 2.2 Photothermal Conversion Properties of Compounds 1–3

To evaluate the potential of compounds **1** to **3** as photothermal conversion materials, their temperature responses under NIR (850 nm) irradiation were examined (Figure 2a). As the irradiation power increased from 0.05–0.3 W cm^−^^2^, the maximum (steady‐state) temperature rose markedly, indicating that light‐to‐heat conversion depends on irradiation intensity (Figure 2b). The temperature profile over time shows that, once irradiation starts, all compounds’ temperatures increase rapidly and reach a quasi‐steady state within a short period (Figure 2c). UV‐treated samples (**1_UV**, **2_UV**, **and 3_UV**) consistently reach higher temperatures than their untreated counterparts, suggesting that UV‐induced states enhance photothermal conversion. This correlates with the diffuse‐reflectance spectra (Figure 1g–i), which reveal improved NIR absorption after UV treatment. The photothermal conversion prior to UV irradiation indicates that electron transfer can occur even under ambient (natural) light. Additionally, cycling tests with repeated ON/OFF irradiation demonstrated highly reproducible temperature responses with only minor decreases in peak temperature, confirming that both the photoinduced state and the materials remain highly stable during thermal cycling (Figure 2d). The photothermal conversion efficiencies were calculated using the method described in the Supporting Information (Figures S10 and S11 and Table S1). The photothermal conversion efficiencies of compounds **1–3** were determined to be 15.0%, 24.5%, and 20.7%, respectively. Although the present materials were not specifically optimized as solar evaporators, these efficiencies are reasonable for molecular crystalline photothermal conversion materials and clearly demonstrate the contribution of UV‐induced radical states to enhanced NIR‐to‐heat conversion [7, 8]. Because **2** exhibited the highest photothermal conversion efficiency, stable photothermal cycling behavior, and relatively low reflectance in the NIR region, **2_UV** was selected as a representative material for subsequent experiments in solar steam generation and seawater desalination.

**FIGURE 2:**
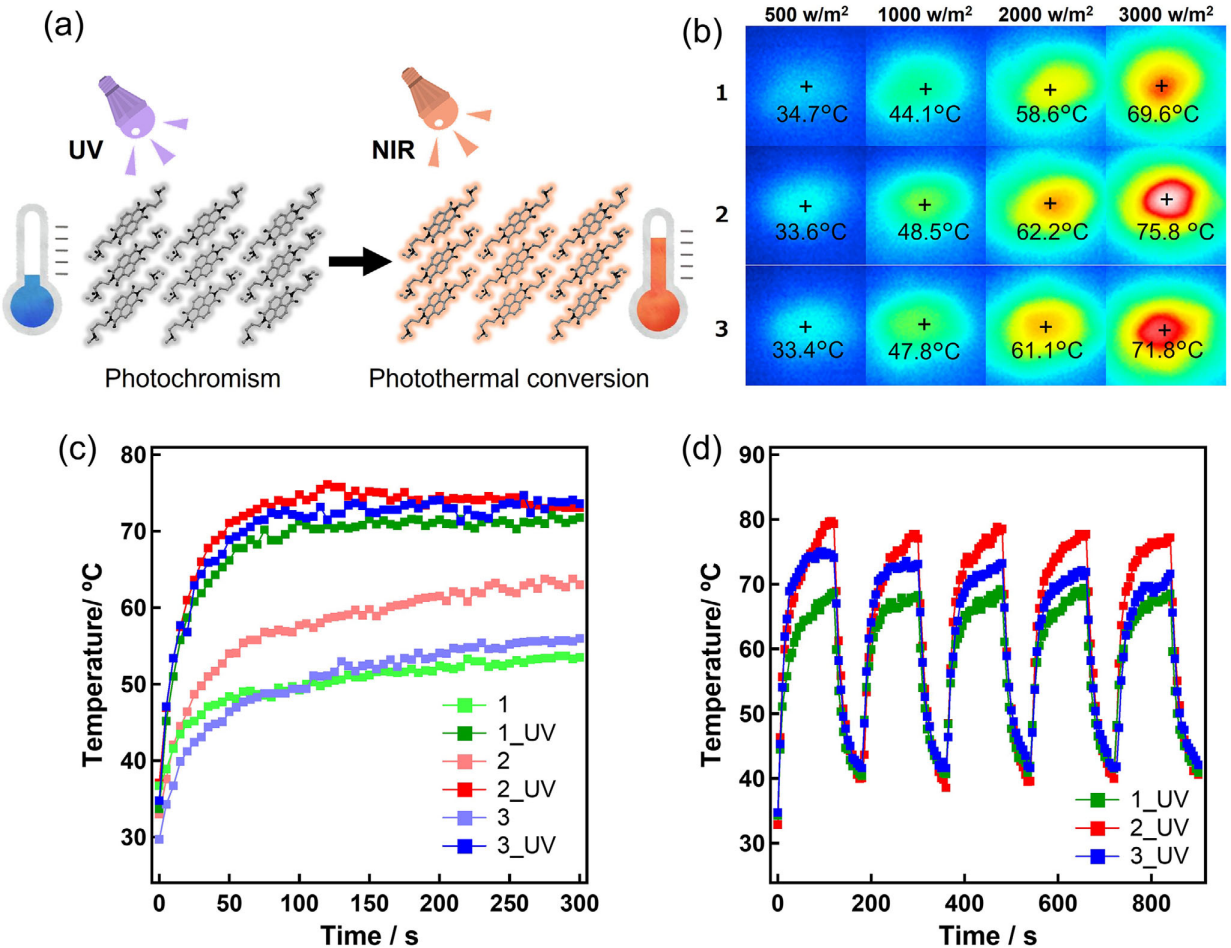
(a) Schematic illustration of UV‐induced photochromism and NIR‐driven photothermal conversion. (b) Thermal camera images showing irradiation‐intensity dependence. (c) Photothermal conversion of the sample before and after UV irradiation under NIR illumination at 0.3 W cm^−^^2^. (d) Repeated measurements under ON–OFF cycles of NIR irradiation.

### 2.3 Solar‐Driven Vapor Generation

A fixed amount of powder sample **2_UV**, which had been adequately pre‐irradiated with UV light (365 nm), was placed in a wooden container made of Japanese cedar (inner diameter: 4.7 cm) and floated on the surface of 100 mL of water in a 200 mL beaker (Figure 3a). The sample was then exposed to a solar simulator (1 sun), and the amount of water evaporated was measured by the change in water mass before and after irradiation (Figure 3b). Under 1 sun irradiation, the surface temperature of **2_UV** increased from 24°C to 37.4°C, indicating that evaporation would be facilitated (Figure 3c).

**FIGURE 3:**
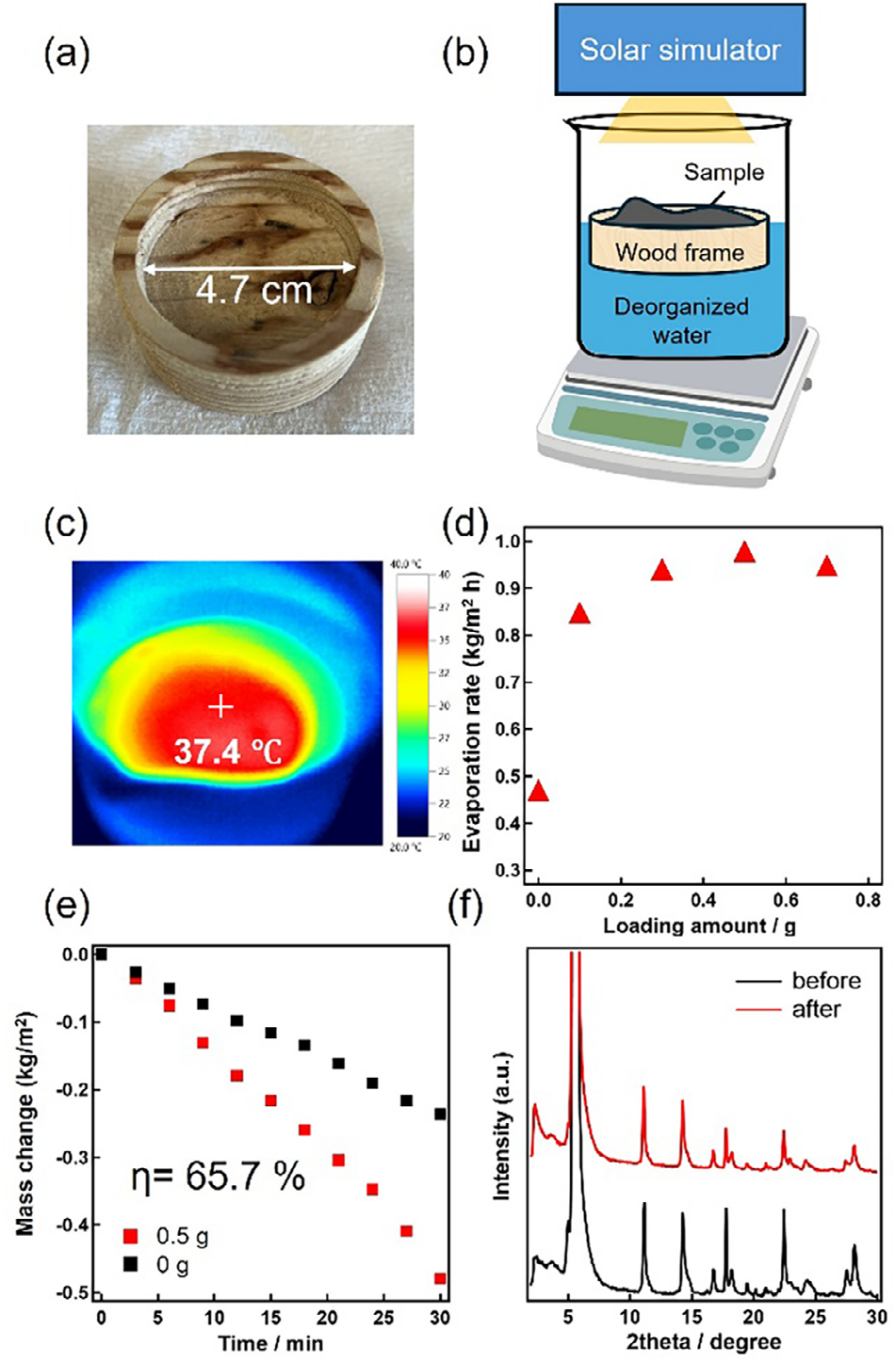
(a) Wooden container used in the experiment. (b) Schematic diagram of the evaporation experiment. (c) Surface temperature of **2_UV** under 1 sun irradiation, recorded with a thermal imaging camera. (d) Sample‐amount dependence of the evaporation rate. (e) Time‐dependent water evaporation over time. (f) PXRD patterns of **2_UV** before and after the water evaporation experiment.

To evaluate the evaporation of pure water as a function of sample loading, tests were conducted using powders of different masses under 1 solar irradiation for 1 h. The highest evaporation rate was achieved using 0.5 g of **2_UV** under the conditions of this experiment. Increasing the loading amount actually decreased the evaporation, indicating that excessive loading hindered the process (Figure 3d). The evaporation rate reached 0.980 kg·h^−^^1^·m^2^, which is 2.13 times higher than the rate (0.461 kg·h^−^^1^·m^2^) measured under the same conditions without loading compounds into wooden containers. (Figure 3e). The evaporation efficiency (*η*) was calculated as the fraction of incident optical energy used for the water's liquid‐vapor phase change based on the evaporation experiment data. As shown in previous reports, *η* was defined as follows:

$$\eta =\frac{\dot{m}\;h_{lv}}{C_{\mathrm{opt}}\;q_{i}}$$

where $\dot{m\;}$ is the evaporation rate normalized by the irradiation area (kg∙m^−^^2^∙s^−^^1^), $h_{lv}$ is the enthalpy of liquid‐to‐vapor phase transition, $C_{\mathrm{opt}}$ is the optical concentration factor, and $q_{i}$ is the incident solar intensity. In this study, *h_lv_* = 2.406 × 10^6^ J·kg^−1^ was used as the value corresponding to the measured surface temperature. Since the experiments were conducted under 1 sun conditions, *C*_opt_ = 1 and *q_i_* = 1000 W·m^−2^ were assumed, and the evaporation efficiency was calculated to be 65.5%. This value falls within the range reported for organic photothermal evaporators, though it is not among the highest achieved with optimized porous supports such as PU foams or hydrogels (Table S2). This result indicates that compound **2** can convert incident solar energy into heat and enhance interfacial water evaporation even in a simple wood‐supported configuration. PXRD measurements were taken to confirm the structural stability before and after the experiment. As a result, there were no significant changes in the diffraction pattern before and after the experiments, indicating that the crystalline structure of compound **2** remained intact throughout the evaporation process (Figure 3f).

The desalination performance of natural seawater was assessed by measuring the photothermal material's solar‐driven steam‐generation capability, as shown in the experimental setup in Figure 4a. Natural seawater (100 mL) from Tokyo Bay was placed in a 200 mL beaker with a wooden frame holding **2_UV** floating on the water surface, and a solar simulator for irradiation was assembled. This setup was positioned inside a 500 mL beaker, with the top tightly covered with plastic wrap to prevent steam from escaping. During the experiment, the evaporated water was collected, and the metal ions (Na^+^, K^+^, Mg^2^^+^, and Ca^2^^+^) present were analyzed using ICP‐OES (Figure 4b). These trace metal ions may have been coevaporated with the water as there were or contaminated by used experimental devices, but the water is considered safe to drink because the ion concentrations are below the WHO drinking water guideline. Compound **2** also retained its structure after immersion in seawater for 72 h, indicating its high stability in seawater (Figure S12). Based on these results, the steam generation system using compound **2_UV** effectively evaporates seawater, indicating that further research is necessary to develop materials with higher desalination efficiency.

**FIGURE 4:**
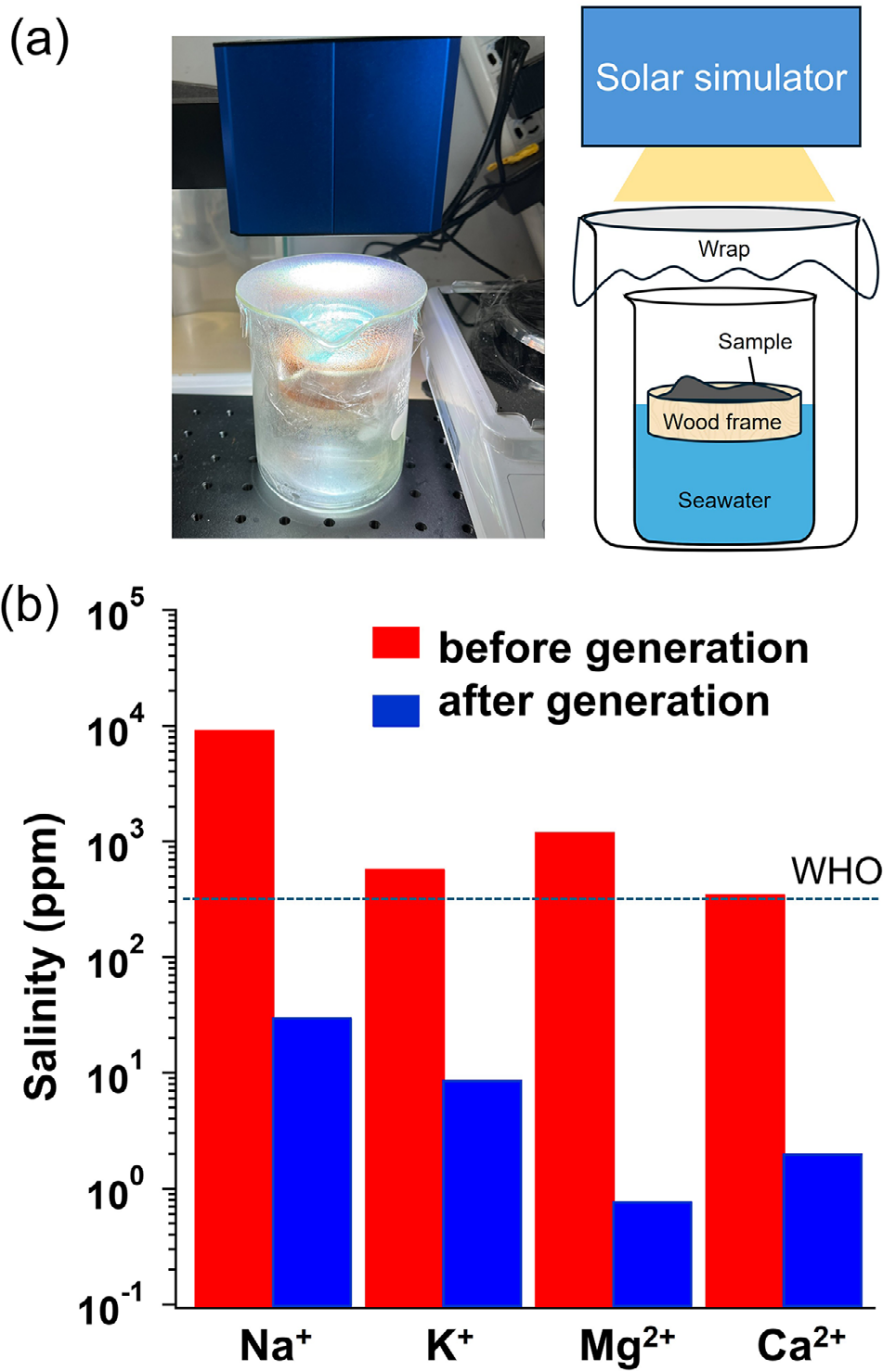
(a) Photograph and schematic illustration of the seawater desalination experiment using **2_UV**. (b) Ion concentrations of seawater and the collected water obtained from the evaporation experiment.

## 3 Conclusion

In summary, donor–acceptor naphthalenediimide molecules bearing tertiary amine donors act as effective organic photothermal materials through photochromism‐induced radical formation. UV irradiation creates stable electron‐transfer states that boost near‐infrared absorption and enable rapid photothermal heating. The most efficient compound exhibits a photothermal conversion efficiency of 24.5% and remains stable over multiple irradiation cycles. When integrated into a floating evaporation platform, the material achieves a water evaporation rate of 0.98 kg m^−^^2^ h^−^^1^ and an energy conversion efficiency of 65.5% under 1 sun illumination. Desalination tests with natural seawater confirm effective salt rejection. These results demonstrate that neutral photochromic donor–acceptor molecules are promising candidates for customizable organic photothermal materials for solar‐driven water purification.

## Conflicts of Interest

The authors declare no conflicts of interest.

## Supporting information




**Supporting File**: chem71482‐sup‐0001‐SuppMat.docx

## Data Availability

The data that supports the findings of this study are available in the Supporting Information of this article
