# Supplementary material for: Photochromic Donor–Acceptor Naphthalenediimides Enabling Near‐Infrared Photothermal Conversion for Seawater Desalination
Source: Chemistry. 2026 Jul 31;32(40):e71482. doi: 10.1002/chem.71482 (PMC13716016; doi:10.1002/chem.71482)
Supplement: Supplementary file 1 [file CHEM-32-e71482-s001.docx]

**Supplementary Information**

Photochromic Donor-Acceptor Naphthalenediimides Enabling Near-Infrared Photothermal Conversion for Seawater Desalination

Sotaro Kusumoto,^a^* Soushi Kurihara,^a^ Masaya Shimabukuro,^b^ Naoki Ogiwara,^c^ Nobutsugu Hamamoto,^d^ Jack Harrowfield,^e^ Yang Kim,^f^ Yoshihiro Koide^a^*

^a^ Department of Applied Chemistry, Faculty of Chemistry and Biochemistry, Kanagawa University, 3-27-1 Rokkakubashi, Kanagawa-ku, Yokohama 221-8686, Japan.

E-mail: [kusumoto@kanagawa-u.ac.jp](mailto:kusumoto@kanagawa-u.ac.jp).

^b^ Laboratory for Biomaterials and Bioengineering, Institute of Integrated Research, Institute of Science Tokyo, 2-3-10, Kanda-Surugadai, Chiyoda-ku, Tokyo 101-0062, Japan.

^c^ Center of Advanced Instrumental Analysis, Kyushu University, Kasuga-Koen, Kasuga, Fukuoka 816-8580, Japan

^d^ Laboratory for Chemistry and Life Science, Institute of Integrated Research, Institute of Science Tokyo, 4259 Nagatsuta-cho, Midori-ku, Yokohama, Kanagawa 226-8501, Japan.

^e^ ISIS, Université de Strasbourg, 8 allée Gaspard Monge, 67083 Strasbourg, France

^f^ Department of Chemistry, Graduate School of Science and Technology, Kumamoto University, 2-39-1 Kurokami, Chuo-ku, Kumamoto 860-8555, Japan.

Corresponding author: S. Kusumoto

E-mail: [kusumoto@kanagawa-u.ac.jp](mailto:kusumoto@kanagawa-u.ac.jp)

Experimental

Materials and Instruments.

All chemicals and solvents used in the preparation were obtained from Tokyo Kasei Co. and Wako Pure Chemical Industries, Ltd., and used without additional purification. The compounds **1**-**3** were prepared following the reported method with minor modifications.^1^

Syntheses of 1-3

**1**

Naphthalene-1,4,5,8-tetracarboxylic dianhydride (0.67 g, 2.5 mmol) and [*N*,*N*-Dimethyl-1,3-propanediamine](https://www.tcichemicals.com/JP/ja/p/D0790) (0.51 g, 5.0 mmol) were dissolved in DMF (20 mL), and the mixture was heated at 100 °C with stirring for 24 hours. After cooling to room temperature, the reaction solution was allowed to stand for an additional 24 hours, during which crystals precipitated. The resulting crystals were collected by suction filtration to yield **1** as crystalline solids (0.97 g, 95.1%). Calcd for C_24_H_28_N_4_O_4_, C, 66.04; H, 6.47; N, 12.84. Found C, 65.94; H, 7.17; N, 13.20%.

**2**

Compound **2** was prepared following the procedure for **1**. Naphthalene-1,4,5,8-tetracarboxylic dianhydride (0.58 g, 2.5 mmol) and *N*,*N*-diethylethylenediamine (0.67 g, 5.0 mmol) were dissolved in DMF (20 mL) and heated at 100 °C for 24 hours. After cooling to room temperature, the mixture was left to stand for 24 hours, and the resulting crystals were collected by suction filtration, affording **2** (0.90 g, 77.1%). Calcd for C_26_H_32_N_4_O_4_, C, 67.22; H, 6.94; N, 12.06. Found C, 67.46; H, 7.44; N, 12.10%.

**3**

Compound **3** was prepared following the procedure for **1**. Naphthalene-1,4,5,8-tetracarboxylic dianhydride (0.58 g, 2.5 mmol) and 1-(3-aminopropyl)pyrrolidine (0.68 g, 5.0 mmol) were dissolved in DMF (20 mL) and heated at 100 °C for 24 hours. After cooling to room temperature, the mixture was left to stand for 24 hours, and the resulting crystals were collected by suction filtration, yielding **3** (1.0 g, 83.8%). Calcd for C_28_H_32_N_4_O_4_, C, 68.83; H, 6.60; N, 11.47. Found C, 68.81; H, 6.83; N, 11.27%.

Physical measurements

Elemental analyses were performed with an Elementar Vario Micro Cube analyzer. PXRD data were collected using a RIGAKU RINT-Ultima III X-ray diffractometer (40 kV/40 mA) using Cu Kα radiation (λ = 1.5406 Å), covering a 2*θ* range of 2–30° with a step width of 1.0°. Diffuse reflectance spectra were obtained with a SHIMADZU UV-2600i Plus equipped with an integrating sphere unit ISR-2600Plus. Photothermal conversion data were performed using a Testo-871. Inductively coupled plasma optical emission spectroscopy (ICP–OES) measurements were performed using a PerkinElmer Avio 220 Max.

Solar steam generation experiment

The samples were irradiated under 1 sun using a solar simulator (Ossila Ltd., G2009B1). The powder sample (0.1, 0.3, 0.5, or 0.7 g) was thinly and uniformly spread in a cylindrical Japanese cedar container (outer diameter: 6.0 cm, inner diameter: 4.7 cm, height: 2.4 cm, and inner depth: 0.8 cm). During irradiation, the mass of water in the beaker was recorded in real time using an electronic balance. The sample temperature was measured using a thermal imaging camera (testo 871). The irradiation lasted for 30 min, and the change in water mass was monitored over time.

To evaluate the applicability of 2_UV to practical solar-driven seawater desalination, natural seawater (100 mL) collected from Tokyo Bay was placed in a 200 mL beaker. A wooden frame supporting floating 2_UV was placed on the water surface, and the system was irradiated using the solar simulator. The setup was then placed inside a 500 mL beaker, and the top was tightly sealed with plastic wrap to prevent water vapor from escaping. During irradiation, the evaporated water was collected, and the concentrations of metal ions (Na⁺, K⁺, Mg²⁺, and Ca²⁺) in the collected water were analyzed by ICP-OES.

Computational methods

First-principles calculations were performed using the projector-augmented wave (PAW) method^1^ as implemented in the Vienna Ab initio Simulation Package (VASP)^2,3^ to optimize crystal structures. We adopted the vdW-DF2 functional as the exchange-correlation functional.^4^ The cutoff energy for the plane-wave basis set was set to 800 eV. Γ-centered Monkhorst–Pack k-point meshes of 4 × 3 × 2 were used for the geometry optimization and electronic structure calculations of **1**–**3**.

**Fig. S1** PXRD patterns of (a) **1**, (b) **2,** and (c) **3**. Simulated patterns from single-crystal X-ray diffraction data (black), pristine samples (red), UV-irradiated samples after 5 min (blue), and NIR-irradiated samples after 5 min at 0.3 W cm⁻² (green).

**Fig. S2** Crystal packing structures of **1-3** viewed along the *a*-axis.

**Fig. S3** Centroid-to-centroid distances between adjacent NDI cores in **1** (a), **2** (b), and **3** (c).

**Fig. S4** 　The shortest distance between the N-donor and the NDI acceptor moiety in **1-3**.

**Fig. S5** Electrostatic potential (ESP) surface maps of compounds **1**–**3** viewed from the top and side directions. The ESP distributions show electron-rich tertiary amine moieties and electron-deficient NDI cores, supporting the donor–acceptor character of these molecules.

**Fig. S6** Kubelka–Munk spectra of **1** (a), **2** (b), and **3** (c) before (red) and after (black) UV irradiation (λ = 365 nm, 0.03 W cm⁻²) for 5 min.

**Fig. S7** ESR spectra of compound **2** before and after UV irradiation.

**Fig. S8** Density of states (DOS) and electron density distributions of the VBM and CBM for **1** (a), **2** (b), and **3** (c). The localized nature of the VBM results in relatively flat valence bands near the Fermi level.

**Fig. S9** Time-dependent diffuse reflectance spectra of **1** (a), **2** (b), and **3** (c).

**Calculation of Photothermal Conversion Efficiency**

To assess the photothermal conversion efficiency of each compound, the specific heat capacity was measured with a differential scanning calorimeter (DSC), and the temperature decay was recorded after NIR irradiation was stopped (Figure S6). For analyzing the temperature decay curves, the dimensionless temperature difference was defined using the following equation^8^,

$$\theta=(T-T\_surr)/(T\_max-T\_surr )$$

and ln (𝜃) was plotted as a function of time. The slope was determined from the linear fit. The specific heat capacities were measured using DSC: *C_p_* = 1463.9 J K^-1^ kg^-1^ for **1**, 1546.956 J K^-1^ kg^-1^ for **2**, and 1279.368 J K^-1^ kg^-1^ for **3**. The photothermal conversion efficiency ($\eta$) was calculated using the following equation:

$$\eta=\frac{hS\left( T_{\max} - T_{\mathrm{surr}} \right)}{I(1-R)}$$

where $hS$is the product of the heat transfer coefficient ($h$) and the surface area ($S$) of the sample, which was determined by

$$hS=-\frac{mC_{p}}{\text{slope}}$$

Here, $m$is the mass of the sample, and the “slope” is obtained from the linear fit of $\ln(\theta)$versus time (Fig. S6). The photothermal conversion efficiencies calculated using these equations are 15.0% for **1**, 24.5% for **2**, and 20.7% for **3**, respectively (Table S1).

**Fig. S10** DSC curves of the blank pan, sapphire standard, and samples **1**–**3** used for the determination of heat capacity by the sapphire method.

**Fig. S11** Cooling decay curves after switching off NIR irradiation for **1** (a, d), **2** (b, e), and **3** (c, f), and the corresponding time vs ln(θ) plots.

**Table S1** Parameters used to calculate the photothermal conversion efficiency.

|  | **1** | **2** | **3** |
| --- | --- | --- | --- |
| Maximum temperature (T_max_ ℃) | 69.6 | 86.6 | 75.8 |
| Ambient temperature (T_surr,_ ℃) | 31.2 | 31.3 | 31 |
| NIR irradiation intensity (W/m²) | 2924 | 2924 | 2924 |
| Reflectance at 850 nm (R %) | 21.95 | 20.55 | 24.21 |
| Sample mass (m, g) | 0.029 | 0.03 | 0.032 |
| Specific heat capacity (Cp, J kg⁻¹ K⁻¹) | 1463.9 | 1546.956 | 1279.368 |
| Slope of the ln(θ)–time plot (slope) | -39.708 | -45.444 | -36.776 |
| Photothermal conversion efficiency (η %) | 15.0 | 24.5 | 20.7 |

**Table S2** Selected photothermal conversion materials and their solar-driven water evaporation performance

| Material | **solar intensity**  **(kW m^–2^)** | **efficiency (%)** | **evaporation rate (kg m^–2^ h^–1^)** | **reference** |
| --- | --- | --- | --- | --- |
| **croconium derivative**  **(**CR-TPE-T) | 1 | 87.2 | **1.272** | 6 |
| ABTS^+・^ | 1 | 97.0 | 1.407 | 7 |
| PPy origami | 1 | 91.5 | 2.12 | 8 |
| DDPA-PDN cellulose paper | 1 | 73.98 | 1.07 | 9 |
| 4OCSPC (phthalocyanine derivative) | 1 | 86.6 | 1.262 | 10 |
| TPyP+PU foam | 1 | 56 | 0.81 | 11 |
| graphene oxide (GO) film | 1 | 80 | 1.45 | 12 |
| Photochromic NDI derivative | 1 | 65.5 | 0.98 | This work |

**Abbreviations:** ABTS, 2,2′-azinobis(3-ethylbenzothiazoline-6-sulfonic acid) radical cation; PPy, polypyrrole; DDPA-PDN, 2,17-bis(diphenylamino)dibenzo[a,c]naphtho[2,3-*h*]phenazine-8,13-dione; PU, polyurethane; TpyP, tetrapyridylporphyrin; NDI, naphthalene diimide.

**Fig. S12** PXRD patterns of **2** before and after immersion in seawater for 72 h.

[1]	Y. Matsunaga, K. Goto, K. Kubono, K. Sako, T. Shinmyozu, “Photoinduced Color Change and Photomechanical Effect of Naphthalene Diimides Bearing Alkylamine Moieties in the Solid State”, *Chem. Eur. J.* **2014**, *20*, 7309–7316.

[2]	P. E. Blöchl, “Projector Augmented-Wave Method”, *Phys. Rev. B* **1994**, *50*, 17953–17979.

[3]	G. Kresse, J. Furthmüller, “Efficient Iterative Schemes for Ab Initio Total-Energy Calculations Using a Plane-Wave Basis Set”, *Phys. Rev. B* **1996**, *54*, 11169–11186.

[4]	G. Kresse, D. Joubert, “From Ultrasoft Pseudopotentials to the Projector Augmented-Wave Method”, *Phys. Rev. B* **1999**, *59*, 1758–1775.

[5]	K. Lee, É. D. Murray, L. Kong, B. I. Lundqvist, D. C. Langreth, “Higher-Accuracy van der Waals Density Functional”, *Phys. Rev. B* **2010**, *82*, 081101.

[6] G. Chen, J. Sun, Q. Peng, Q. Sun, G. Wang, Y. Cai, X. Gu, Z. Shuai, B. Z. Tang, Biradical-Featured Stable Organic-Small-Molecule Photothermal Materials for Highly Efficient Solar-Driven Water Evaporation, *Adv. Mater.* **2020**, *32*, 1908537.

[7] J. Xu, Q. Chen, S. Li, J. Shen, P. Keoingthong, L. Zhang, Z. Yin, X. Cai, Z. Chen, W. Tan, Charge-Transfer Cocrystal via a Persistent Radical Cation Acceptor for Efficient Solar-Thermal Conversion, *Angew. Chem. Int. Ed.* **2022**, *61*, e202202571.

[8] W. Li, Z. Li, K. Bertelsmann, D. E. Fan, Portable Low-Pressure Solar Steaming-Collection Unisystem with Polypyrrole Origamis, *Adv. Mater.* **2019**, *31*, 1900720.

[9] Y. Cui, J. Liu, Z. Li, M. Ji, M. Zhao, M. Shen, X. Han, T. Jia, C. Li, Y. Wang, Donor–Acceptor-Type Organic-Small-Molecule-Based Solar-Energy-Absorbing Material for Highly Efficient Water Evaporation and Thermoelectric Power Generation, *Adv. Funct. Mater.* **2021**, *31*, 2106247.

[10] X. Han, Z. Wang, M. Shen, J. Liu, Y. Lei, Z. Li, T. Jia, Y. Wang, A Highly Efficient Organic Solar Energy-Absorbing Material Based on Phthalocyanine Derivative for Integrated Water Evaporation and Thermoelectric Power Generation Application, *J. Mater. Chem. A* **2021**, *9*, 24452–24460.

[11] Y. Zhang, H. Yan, X. Wang, Z. Zhang, F. Liu, S. Tu, X. Chen, Highly Efficient Solar-Absorber Composite Material Based on Tetrapyridylporphyrin for Water Evaporation and Thermoelectric Power Generation, *RSC Adv.,* **2022**, *12*, 28997-29002.
